# ﻿The subfamily Dermestinae (Coleoptera, Dermestidae) from Saudi Arabia

**DOI:** 10.3897/zookeys.1138.90338

**Published:** 2023-01-05

**Authors:** Jiří Háva, Mahmoud S. Abdel-Dayem, Hathal M. Aldhafer

**Affiliations:** 1 Private Entomological Laboratory and Collection, Rýznerova 37/37, CZ-252 62 Únětice u Prahy, Prague-West, Czech Republic Private Entomological Laboratory and Collection Prague-West Czech Republic; 2 King Saud University Museum of Arthropods (KSMA), Plant Protection Department, College of Food and Agricultural Sciences, King Saud University, P.O. Box 2460 Riyadh 11451, Saudi Arabia King Saud University Riyadh Saudi Arabia; 3 Entomology Department, Faculty of Science, Cairo University, Giza, 12613, Egypt Cairo University Giza Egypt

**Keywords:** Beetles, Dermestini, distribution, fauna, Marioutini, new records

## Abstract

In this study, the fauna of Saudi Arabian Dermestinae (Coleoptera, Dermestidae) is summarised. Six *Dermestes* species and single species from two Marioutini genera, *Mariouta and Rhopalosilpha*, are reported. Dermestes (Dermestinus) undulatus Brahm, 1790 and Dermestes (Dermestes) haemorrhoidalis Küster, 1852 are newly recorded from Saudi Arabia. A list of Dermestinae species from the Arabian Peninsula is provided with their distributions.

## ﻿Introduction

Dermestinae is a subfamily of Dermestidae with a worldwide distribution, but concentrated in the Holarctic and Afrotropical areas. According to Háva (2015, 2022), there are approximately 95 species assigned to only five genera under two tribes: Dermestini with three genera, *Derbyana* Lawrence & Ślipiński, *Dermalius* Háva, and *Dermestes* Linnaeus, 1758, and Marioutini with two genera, *Mariouta* Pic and *Rhopalosilpha* Arrow; additionally there is the fossil tribe Paradermestini with one genus, *Paradermestes* Deng, Ślipiński, Ren & Pang (Háva 2015, 2022). The genus *Dermestes* is the largest genus in Dermestinae and recently included 89 species and subspecies worldwide (Háva 2015, 2022). Members of the subfamily are generally recognised by their elongate body structure, lack of ocelli, and males with small tufts of erect setae on the abdominal ventrites (females are without tufts). Larvae are zoonecrophagous.

The first data concerning Dermestinae of Saudi Arabia date back to the second half of the 20^th^ century. In the early 1960s, the Egyptian entomologist F. Shalaby (1961) was perhaps the first who catalogued data on *Dermestesmaculatus* DeGeer, 1774. The work of the Polish entomologist M. Mroczkowski (1979) was the first important faunistic study on the Saudi Arabian Dermestidae fauna. His work was based on the collection made by W. Büttiker who intensively explored many areas of Saudi Arabia, and he recorded three *Dermestes* species. Mroczkowski and Ślipiński (1997) published their review and keys to world genera and species of the tribe Marioutini and reported *Marioutastangei* Reitter, 1910 and *Rhopalosilphawasmanni* Arrow, 1929 from Saudi Arabia.

From the beginning of the 21^st^ century and during the last two decades, the forensic importance of dermestid beetles attracted the attention of many workers from Saudi Arabia (e.g., Abouzied 2014; Alajmi et al. 2016; Al-Shareef and Al-Mazyad 2017; Al-Shareef and Zaki 2017; Mashaly 2017; Shaalan et al. 2017; Mashaly et al. 2018, 2019; Al-Dakhil and Alharbi 2020; Al-Qahtni et al. 2020). However, the faunistic data on Dermestinae were published as part of general surveys of insects or beetles (Abdel-Dayem et al. 2017, 2020; Elgharbawy 2018). The systematic, faunistic, and distribution of Dermestinae in Saudi Arabia are still not well known, and few works have been published. This paper aims to summarise the known Saudi Arabian Dermestinae and update distribution data.

## ﻿Materials and methods

The data on the distribution of the species in the subfamily Dermestinae (Coleoptera, Dermestidae) in Saudi Arabia is based on three main sources. The first are the historical works of Shalaby (1961), Mroczkowski (1979), Mroczkowski and Ślipiński (1997), and additionally the recent publication of Abouzied (2014), Abdel-Dayem et al. (2017, 2020), Al-Shareef and Zaki (2017), Mashaly (2017), Elgharbawy (2018), Mashaly et al. (2019), Al-Dakhil and Alharbi (2020), and Al-Qahtni et al. (2020). The second source are specimens preserved in the insect collections of the King Saud University Museum of Arthropods (**KSMA**) in Riyadh, Saudi Arabia, the Florida State Collection and Arthropods (**FSCA**), and the collection of the first author. The third source is an extended field survey conducted by the second and third authors, which is still ongoing. The collected specimens were deposited in the collections of KSMA, unless otherwise indicated (JHAC: Jiří Háva). The nomenclature follows Motyka et al. (2022). A note entry summarises published and current data on the species distribution within Saudi Arabia. The general range and the world distribution data were derived from the catalogues of Háva (2015, 2022).

For each material lot examined, the following label data are provided as follows: Country name (in capital letters) at the beginning. Then each record starts with a bullet point (•) followed by the number of examined specimens followed by sex (if determined) or “ex” (if the specimen sex could not be recognised because the abdomen was lost, damaged, or other reasons); Saudi Province followed by a comma (,), governorate, locality; geographical coordinates; elevation (m), collection date; collector(s) name followed by “leg.”; method of collection (bait trap (**BT**), handpicking (**HP**), light trap (**LT**), malaise trap (**MT**), pitfall trap (**PT**), sweeping net (**SW**)), the identifier name followed by “det.”, and the depository collection acronym. The material examined was arranged in alphabetical order with respect to the Saudi province, governorate, and locality name. Data were then arranged in chronological order according to the month of collection. Records with the same locality data, except for slight differences (such as date of collection, altitude, collector/s), were reported together with the second label, given “same collection data as for preceding” and followed by a semicolon (;) and the different data.

The following acronyms of type depositories are used in the text:

**JHAC** Jiří Háva, Private Entomological Laboratory & Collection, Únětice u Prahy, Prague-West, Czech Republic;

**FSCA** Florida State Collection and Arthropods, Gainesville, USA;

**KSMA** King Saud University Museum of Arthropods, Plant Protection Department, College of Food and Agriculture Sciences, King Saud University, Riyadh, Saudi Arabia.

## ﻿Results

### ﻿Family Dermestidae Latreille, 1804

**Subfamily Dermestinae Latreille, 1804**

**Tribe Dermestini Latreille, 1804**

**Genus *Dermestes* Linnaeus, 1758**

#### Subgenus Dermestes s. str.

##### ﻿ Dermestes (Dermestes) ater

Taxon classificationAnimalia

DeGeer, 1774

2BEFCB1C-5D53-52C4-819F-6665EA145EA3

Fig. 1A, B

###### Material examined.

Saudi Arabia • 1 ♂; Eastern Province, An Nuayriah, Al Sarar; 27°25'45.5"N, 48°27'0.0"E; 60 m a.s.l.; 2 Mar. 2011; H. Al Dhafer; H. Setyaningrum & A. Al Ansi leg.; collected from carcasses on the road;, J. Háva det.; KSMA • 1 ♀; Makkah Province, Jeddah, “Ras Halibah” [Ras Hatibah];, 7 May. 1982; W. Büttiker leg.;JHAC • 4 ex; Riyadh Province, Dirab, Al-Dhab Farm; 5 Oct. 1986; collected from chicken farm waste, J. Háva det.; KSMA; • 1 ex; Riyadh, Al-Wahah Farm; 12 Oct. 1989; J. Háva det.; KSMA.

**Figure 1.**
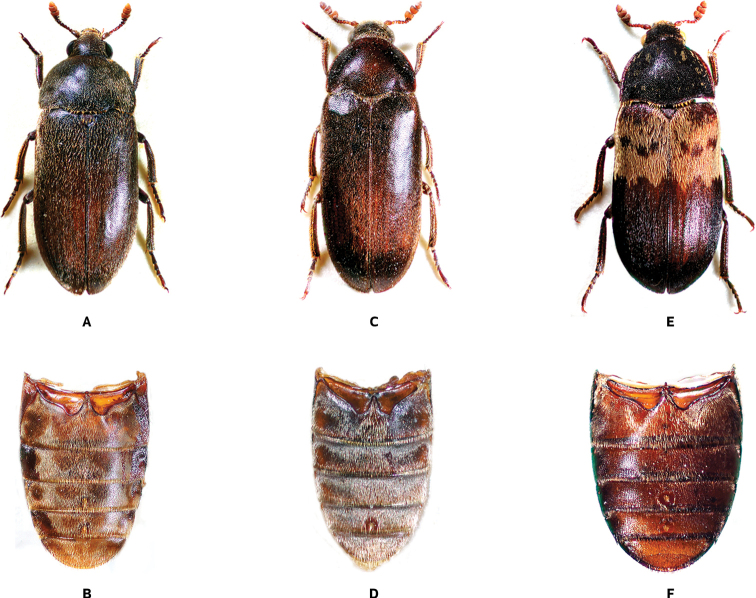
Dorsal habitus and abdominal ventrites (photos by A. Herrmann) of *Dermestes* species **A, B***D.ater* DeGeer, 1774 **C, D***D.haemorrhoidalis* Küster, 1852 **E, F***D.lardarius* Linnaeus, 1758.

###### Note.

This species was previously recorded in Eastern Province at Al Hofuf (Mroczkowski 1979); Dammam (Mroczkowski 1979), Dhahran (Mroczkowski 1979), and Riyadh Province at Riyadh (Mroczkowski 1979). The listed specimens were collected from low elevation areas (<600 m) in central, eastern, and southwestern Saudi Arabia (Fig. 4A).

###### Distribution.

Cosmopolitan (Háva 2007, 2015, 2022).

##### ﻿ Dermestes (Dermestes) haemorrhoidalis

Taxon classificationAnimalia

Küster, 1852

17476388-1179-5E4F-8918-88F319530C16

Fig. 1C, D

###### Material examined.

Saudi Arabia • Riyadh Province, 1 ♀; Al Zulfi, Rawdhat Al Sablh; 26°22.429'N, 44°58.241'E; 670 m a.s.l.; 26 Aug. 2015; H. Al Dhafer, M. Abdel-Deyem, A. El Torkey, A. El Gharbawy, & A. Solimanleg leg.; LT; J. Háva det.; KSMA.

###### Note.

The female specimen was collected at a low elevation (670 m) in a sandy area in central Saudi Arabia (Fig. 4A). This represents a new record for Saudi Arabia.

###### Distribution.

Nearly cosmopolitan (Háva 2015, 2022), where it is widely distributed in Europe; North Africa; Africa: Burundi, Congo, Madagascar, South Africa, Tanzania, Zambia; Asia: China (Liaoning), Iran, Japan, Mongolia, Oman, Russia, South Korea, Vietnam; Australia: New Zealand (introduced); North America: USA; South America: Argentina, Bolivia, Brazil, Peru, Uruguay.

##### ﻿ Dermestes (Dermestes) lardarius

Taxon classificationAnimalia

Linnaeus, 1758

6B0E012C-3E86-5C26-9B53-CB4E4AA2F528

Fig. 1E, F

###### Material examined.

Saudi Arabia • 1 ♀; Makkah Province, Jeddah, “Ras Halibah” [Ras Hatibah]; 7 May. 1982; W. Büttiker leg.; JHAC.

###### Note.

Dermesteslardarius was previously reported from Saudi Arabia without a specific locality (Hagstrum and Subramanyam 2009). The only known female representing this species in Saudi Arabia was collected from the coastal area in Jeddah (Makkah Province) (Fig. 4D).

###### Distribution.

Cosmopolitan (Háva 2011, 2015, 2022).

#### Subgenus Dermestinus Zhantiev, 1967

##### ﻿ Dermestes (Dermestinus) frischii

Taxon classificationAnimalia

Kugellan, 1792

B0A4E452-E4FE-5AF2-9C2E-734035CDBEAB

Fig. 2A–C

###### Material examined.

Saudi Arabia • 2 ♂; Baha Province, Al Mandaq, Amadan; 20°12'11"N, 41°13'43"E; 14 Oct. 2010; H.Aldhafer & H.Fadl leg.; M.S. Abdel-Dayem det.; KSMA • 1 ex; Asir Province, Bareq, Thloth Al Mandhar, Wadi Baqrah; 18°47.476'N, 41°56.310'E; 331 m a.s.l.; 20 Apr. 2011; H. Fadl & H. Setyaningrum leg.; LT;M.S. Abdel-Dayem det.; KSMA • 1 ♀; Eastern Province, Al Jubail, Ras al Ghar; 26°15'34"N, 49°52'01"E; 16 Apr. 2010; H. Al Dhafer leg.; HP; J. Háva det.; KSMA • 3 ♂, 4 ♀; Eastern Province, An Nuayriyah, Al Sarar; 27°25'45.5"N, 48°27'00"E; 60 m a.s.l.;, 2 Mar. 2011; H. Al Dhafer, H. Setyaningrum & A. Al Ansi leg.; collected from Carcases; M.S. Abdel-Dayem det.; KSMA • 1 ex; same collection data as for preceding; J. Háva det.; KSMA • 5 ex; Eastern Province, Dammam, near shore; 26°21'3.744"N, 50°13'41.462"E; 3 m a.s.l.; 15 Oct. 2018; A. Alqurashi leg.; PT beside rabbit carcass, M.S. Abdel-Dayem det.; KSMA. • 1 ♂; Makkah Province, Jeddah, Shoiba; 20°51'N, 39°24'E; 1 m a.s.l.; 19 Oct. 1982; W. Büttiker leg.; JHAC • 2 ♂, 1 ♀; Makkah Province, Taif, Al Wesam District; 21°12'17"N, 40°20'43"E; 11 Oct. 2010; H. Al Dhafer, B. Kondratieff, H. Fadl & A. El Gharbawy leg.; M.S. Abdel-Dayem det.; KSMA • 1 ♂, 1 ♀; Riyadh Province, Ad Diriah, Ad Diriah Desert; 6 May. 2010; H. Al Dhafer, A. El Gharbawy & A. El Torkey leg.; MT; M.S. Abdel-Dayem det.; KSMA • 1 ♂, 4 ♀; same collection data as for preceding; Al Amariyah, Animal Production Dept. Farm KSU; 31 Mar. 2008;, LT;M.S. Abdel-Dayem det.; KSMA • 2 ♂, 3 ♀; same collection data as for preceding; Aljabilah, Prince Bander Farm; 26 Apr. 2008; M. Otybi leg.; LT; M.S. Abdel-Dayem det.; KSMA • 1 ♂; same collection data as for preceding; 3 May. 2008; M. Otybi leg.; LT; M.S. Abdel-Dayem det.; KSMA • 1 ♂; same collection data as for preceding; 31 May. 2008; M. Otybi leg.; LT; M.S. Abdel-Dayem det.; KSMA • 1 ♀; same collection data as for preceding; Al Obaiteh, 50 km W. Riyadh, Obikan Farm; 7 May. 2007; M. Otybi leg.; LT; J. Háva det.; KSMA • 1 ♀; same collection data as for preceding; Thonyan Al Thonyan Farm; 28 Jul. 2007; H. Al Ayedh & H. Al Dhafer leg.; LT; M.S. Abdel-Dayem det.; KSMA • 3 ♂, 4 ♀; same collection data as for preceding; Education Farm KSU; 1 Apr. 2008; J. Háva det.; KSMA • 7 ♀; same collection data as for preceding; 2 Apr. 2008; M.S. Abdel-Dayem det.; KSMA • 4 ♂, 6 ♀; same collection data as for preceding; 3 Apr. 2008; M.S. Abdel-Dayem det.; KSMA • 14 ex; same collection data as for preceding; 5 Apr. 2008, M.S. Abdel-Dayem det.; KSMA • 7 ♂, 8 ♀; same collection data as for preceding; 7 Apr. 2008; M.S. Abdel-Dayem det.; KSMA • 2 ♂, 6 ♀; same collection data as for preceding; 9 Apr. 2008; M.S. Abdel-Dayem det.; KSMA • 1 ♂, 11 ♀; same collection data as for preceding 11 Apr. 2008; M.S. Abdel-Dayem det.; KSMA • 1 ♀; same collection data as for preceding; 20 Apr. 2011; H. Setyaningrum leg.; J. Háva det.; KSMA • 1 ♂; Riyadh Province, Alkharj, Al-Shahwan Farm; 24 Mar. 2010; A. Al-Hasbel leg.; SW; J. Háva det.; KSMA • 1 ♀; Riyadh Province, Huraymala, Wadi Huraymala; 770 m a.s.l.; 24 Nov. 1988; C.W. Mills leg.; J. Háva det.; FSCA • 1 ♀; Riyadh Province, Mozahmiya, Al Khararah; 24°24'21"N, 46°14'40"E; 17 Apr. 2012; H. Al Dhafer, H. Fadl, A. El Torkey, M. Abdel-Dayem & A. Al Ansi leg.; LT; M.S. Abdel-Dayem det.; KSMA • 1 ♂, 1 ♀; Riyadh Province, Rumah, Rawdhat khorim; 29 Apr. 2011; Y. Aldryhim leg.; LT; M.S. Abdel-Dayem det.; KSMA • 1 ex; same collection data as for preceding; 25°25.943'N, 47°13.863'E; 572 m a.s.l.; 6 Mar. 2012; PT; M.S. Abdel-Dayem det.; KSMA • 1 ex; same collection data as for preceding; 27 May. 2012; LT; M.S. Abdel-Dayem det.; KSMA.

**Figure 2.**
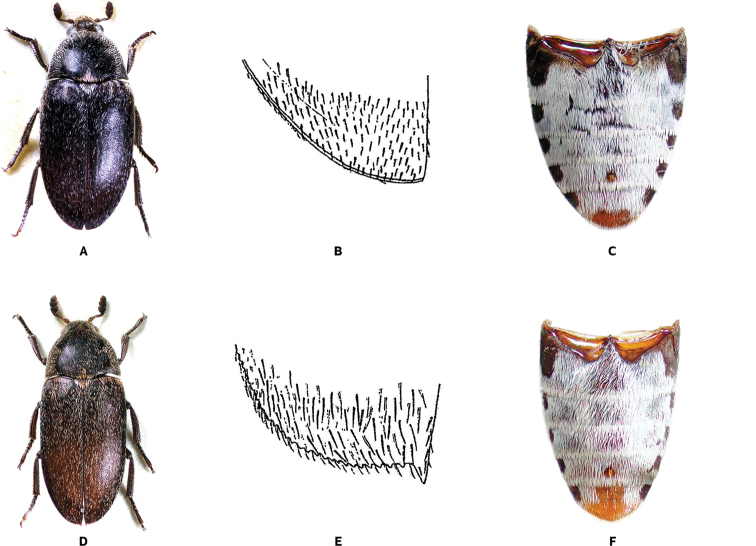
Dorsal habitus, apical part of elytron and abdominal ventrites (photographs by A. Herrmann) of *Dermestes* species. **A–C***D.frischii* Kugellan, 1792 **D–F***Dermestesmaculatus* DeGeer, 1774.

###### Note.

Mroczkowski (1979) documented this species in Jeddah. Recently it was collected at Jeddah from rabbit carcasses (Al-Shareef and Al-Mazyad 2017) and human remains (Al-Shareef and Zaki 2017), and at Riyadh from camel, dog, and goat carcasses (Mashaly et al. 2019) and human corpses (Alajmi et al. 2016). This species has also been collected from sheep carcasses in Riyadh, Jazan, and Arar (Mashaly et al. 2018). The listed specimens were collected at different elevations (7–1920 m) in the central, east, and lowlands and mountainous areas of southwest Saudi Arabia (Fig. 4B).

###### Distribution.

Cosmopolitan (Háva 2007, 2015, 2022).

##### ﻿ Dermestes (Dermestinus) maculatus

Taxon classificationAnimalia

DeGeer, 1774

1B018984-6262-5909-8B37-2721308EFCB0

Fig. 2D–F

###### Material examined.

Saudi Arabia • 2 ♀; Baha Province, Al Mandaq, Amadan; 20°12'11"N, 41°13'43"E; 14 Oct. 2010; H. Al Dhafer, B. Kondratieff, H. Fadl & A. El Gharbawy leg.; J. Háva det.; KSMA • 4 ♀; same collection data as for preceding; 14 Oct. 2010; H.Aldhafer & H.Fadl leg.; J. Háva det.; KSMA • 12 ex; Baha Province, Al Baha, Al-Baher Mountain; 15 Mar. 2010; J. Háva det.; KSMA • 1 ♀; Asir Provence, Khamis Mushayt; 2050 m a.s.l.; 9 Jan. 1998; J. Háva det.;, JHAC • 1 ex; Makkah Province; Taif; 21°12'17"N, 40°20'43"E; 11 Oct. 2010; H. Al Dhafer, B. Kondratieff, H. Fadl & A. El Gharbawy leg.; M.S. Abdel-Dayem det.; KSMA • 6 ex; Eastern Province; Dammam, near shore; 26°21'3.744"N, 50°13'41.462"E; 3 m a.s.l.; 15 Oct. 2018, A. Alqurashi leg.; PT beside rabbit carcass; M.S. Abdel-Dayem det.; KSMA • 1 ♀; Riyadh Province, Ad Diriyah, Al Amariyah, Animal Production Dept. Farm KSU; 23 Mar. 2011; H. Setyaningrum leg.; BT; M.S. Abdel-Dayem det.; KSMA • 1 ex; Riyadh Province, Riyadh; Oct.1989; M.S. Abdel-Dayem det.; KSMA • 4 ex; Riyadh Province, Ad Diriyah, Al Amariyah; 28 Jan. 2008; D. Boy Valenza leg., M.S. Abdel-Dayem det.; KSMA • 2 ex; same collection data as for preceding; Albeer Farm; 29 Oct. 2008; A. Al-Ahmari leg.; SW; M.S. Abdel-Dayem det.; KSMA • 1 ex, same collection data as for preceding; 8 Dec. 2010; SW; M.S. Abdel-Dayem det.; KSMA • 2 ♀; Riyadh Province, Ad Diriyah, Education Farm KSU; 1 Apr. 2008; J. Háva det.; KSMA • 1 ex; same collection data as for preceding; 31 Mar. 2008; M.S. Abdel-Dayem det.; KSMA • 1 ex; same collection data as for preceding; 2 Apr. 2008; M.S. Abdel-Dayem det.; KSMA • 1 ex; same collection data as for preceding; 3 Apr. 2008; M.S. Abdel-Dayem det.; KSMA • 3 ex; same collection data as for preceding; 5 Apr. 2008; M.S. Abdel-Dayem det.; KSMA • 1 ex; same collection data as for preceding; 7 Apr. 2008; M.S. Abdel-Dayem det.; KSMA • 3 ex; same collection data as for preceding; 9 Apr. 2008; M.S. Abdel-Dayem det.; KSMA • 2 ex; same collection data as for preceding; 11 Apr. 2008; M.S. Abdel-Dayem det.; KSMA • 1 ex; same collection data as for preceding; 3 Nov. 2009; H. Setyaningrum leg.; M.S. Abdel-Dayem det.; KSMA • 6 ex; Riyadh Province, Riyadh, Al-Wahah Farm; 15 May. 2021; M.S. Abdel-Dayem det.; KSMA • 1 ex; Riyadh Province, Shaqra; 21 May. 1978; HP from mill waste; J. Háva det.; KSMA.

###### Note.

Dermestesmaculatus is the most common species within the subfamily Dermestinae in Saudi Arabia. It was previously collected from rabbit carcasses at Baha (Abouzied 2014), Al-Ahsa (Shaalan et al. 2017), t Madinah (Al-Dakhil and Alharbi 2020), Jeddah (Al-Shareef and Al-Mazyad 2017), and Riyadh (Mashaly 2017). It has been collected from sheep carcasses in Jizan and Northern Border (Mashaly et al. 2018). Al-Qahtni et al. (2020) have reported the species from dead human bodies in Riyadh. Also, it was collected by other methods from Makkah Province, Jeddah (Shalaby 1961; Mroczkowski 1979) and Riyadh Province, Dierab (Elgharbawy 2018). The species was collected in both low- and highlands (10–2330 m) in the following provinces: Asir, Baha, Eastern Province, Madinah, Makkah, and Riyadh (Fig. 4C).

###### Distribution.

Cosmopolitan (Háva 2011, 2015, 2022).

##### ﻿ Dermestes (Dermestinus) undulatus

Taxon classificationAnimalia

Brahm, 1790

7588E381-546B-5BF7-B020-75FF5AD69B0E

Fig. 3A, B

###### Material examined.

Saudi Arabia • 1 ♀; Asir Province, Khamis Mushayt; 2050 m a.s.l.; 9 Jan.1998;, J. Háva det.; JHAC.

**Figure 3.**
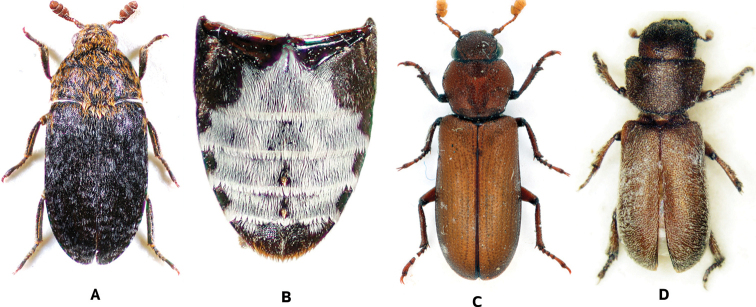
**A, B***Dermestesundulatus* Brahm, 1790 (photographs by A. Herrmann) **A** dorsal habitus **B** Abdominal ventrites **C***Marioutastangei* Reitter, 1910 (photographs by K. Matsumoto) **D***Rhopalosilphawasmanni* Arrow, 1929 (photographs by J. Háva).

###### Note.

**Figure 4.**
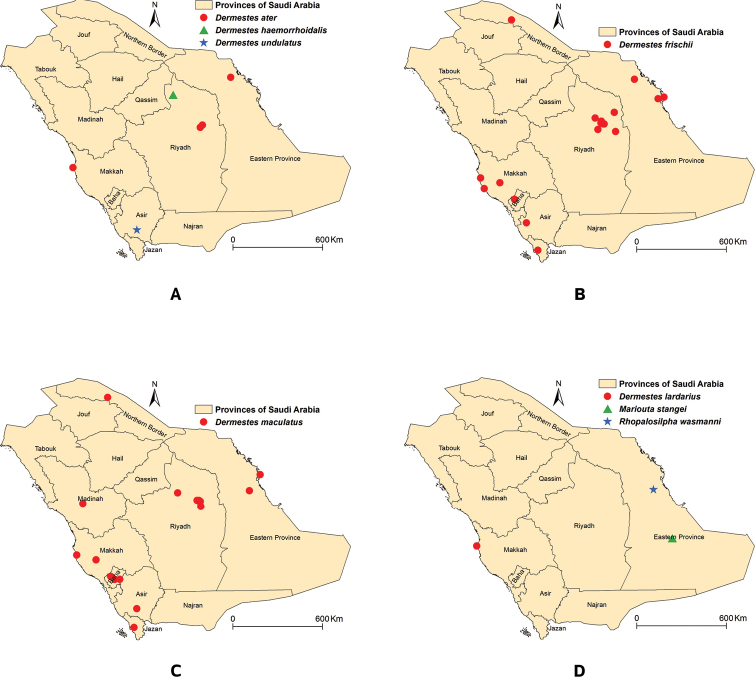
Distribution map of the Dermestinae species in Saudi Arabia **A***Dermestesater*, *D.haemorrhoidalis*, and *D.undulatus***B***D.frischii***C***D.maculatus***D***D.lardarius*, *Marioutastangei*, and *Rhopalosilphawasmanni*.

The only specimen known (a female) was collected from the highlands in southwestern Saudi Arabia (Fig. 4A). This is a new record for Saudi Arabia.

###### Distribution.

Holarctic species (Háva 2015, 2022).

**Tribe Marioutini Jacobson, 1913**

#### Genus *Mariouta* Pic, 1898

##### ﻿ Mariouta stangei

Taxon classificationAnimalia

Reitter, 1910

80E38C90-D052-5C43-A11F-08DE4C6C0F17

Fig. 3C

###### Record.

Saudi Arabia • Eastern Province, Al-Ahsa, Salwah, 248 km S (Rub al Khali) (Mroczkowski and Ślipiński 1997).

###### Note.

This species is only known from a single specimen preserved in the NHMB collection. This specimen was collected by W. Büttiker in May 1985 at a location in the Empty Quarter (Rub al Khali), 248 km south of the town of Salwa in southeastern Saudi Arabia, located near the border with Qatar (Fig. 4D).

###### Distribution.

This taxon is known from the Sultanate of Oman, Pakistan, Saudi Arabia, Sudan, and Turkmenistan (Háva 2015, 2022).

#### Genus *Rhopalosilpha* Arrow, 1929

##### ﻿ Rhopalosilpha wasmanni

Taxon classificationAnimalia

Arrow, 1929

C5E9ECD0-E9F1-5A2F-89DF-A5F9D2F693A8

Fig. 3D

###### Record.

Saudi Arabia • Eastern Province, Hofuf (Mroczkowski and Ślipiński 1997).

###### Note.

Rhopalosilphawasmanni is only known from a single specimen in the NHMB collection. It was collected from Hofuf in eastern Saudi Arabia by W. Büttiker (Mroczkowski and Ślipiński 1997) (Fig. 4D).

###### Distribution.

This very rare species is known only from Iran, Jordan, and Saudi Arabia (Háva 2015, 2022).

## ﻿Discussion

The first forensic case being solved using insects was during the 13^th^ century in China, while the first systematic studies of forensic entomology took place in Germany during the 19^th^ century (Benecke 2001). Despite the earlier published data documenting the Saudi fauna of Dermestinae (Shalaby 1961; Mroczkowski 1979), forensic entomology in this country began only during the last two decades. Accordingly, several studies were conducted during this period in four areas: at the centre of the country (Alajmi et al. 2016; Mashaly 2017; Mashaly et al. 2018, 2019; Al-Qahtni et al. 2020), in the east (Shaalan et al. 2017), in the west (Al-Shareef and Al-Mazyad 2017; Al-Shareef and Zaki 2017; Al-Dakhil and Alharbi 2020), and in the southwest (Abouzied 2014). Taxonomic and faunal studies are needed to support this growing interest in forensic entomology in Saudi Arabia.

During the late stage of decay of animal remains, *Dermestes* species are one of the predominant taxa among forensic insects (Magni et al. 2015). *Dermestesfrischii* and *D.maculatus* have been the most frequently documented dermestid beetles in forensic studies in Saudi Arabia (Alajmi et al. 2016; Mashaly et al. 2018). The current study listed eight species in three genera (*Dermestes*, *Mariouta*, and *Rhopalosilpha*) in two tribes (Dermestini and Marioutini) belonging to the subfamily Dermestinae. *Dermesteshaemorrhoidalis* and *D.undulatus* are recorded for the first time from Saudi Arabia. Based on the world distribution range, the Saudi Dermestinae fauna is dominated by the widespread cosmopolitan or nearly cosmopolitan species, which includes all members of the tribe Dermestini (e.g., *Dermestesater*, *D.haemorrhoidalis*, *D.lardarius*, *D.frischii*, *D.maculatus*, *D.undulatus*; Háva 2015, 2022) (Table 1), while the members of tribe Marioutini, *Marioutastangei* and *Rhopalosilphawasmanni* have a narrower distribution range and appear to have Saharo-Sindian elements (Háva 2015, 2022).

**Table 1.** List of *Dermestes* species from the Arabian Peninsula. Notes: recorded (*) or not recorded (–).

	Kuwait	Saudi Arabia		Yemen		Oman	United Arab Emirates	Qatar
		Farasan Archipelago	Saudi Arabia mainland	Yemen mainland	Socotra Island			
Dermestes (Dermestinus) maculatus DeGeer, 1774	*	–	*	*	*	*	*	*
Dermestes (Dermestinus) frischii Kugellan, 1792	*	–	*	*	*	*	*	*
Dermestes (Dermestinus) undulatus Brahm, 1790	–	–	*	–	–	–	–	–
Dermestes (Dermestes) ater DeGeer, 1774	–	–	*	*	–	*	*	*
Dermestes (Dermestes) haemorrhoidalis Küster, 1852	–	–	*	–	–	–	–	–
Dermestes (Dermestes) lardarius Linnaeus, 1758	–	–	*	*	–	–	*	–

The analysis of data based on the examination of museum specimens and literature records revealed that *D.frischii* and *D.maculatus* are the most abundant and distributed over a fairly wide range in Saudi Arabia. These findings are consistent with what has been documented in several other studies (Shalaby 1961; Mroczkowski 1979; Abouzied 2014; Alajmi et al. 2016; Shaalan et al. 2017; Elgharbawy 2018; Al-Dakhil and Al-Harbi 2020). However, the remaining Dermestinae are rare or very rare species, either documented from a few specimens (e.g., *D.ater*) or a single specimen (e.g., *D.haemorrhoidalis*, *D.lardarius*, *D.undulatus*, *M.stangei*, and *R.wasmanni*). This may be due to different feeding behaviours or a rarity of these species in the Saudi fauna. Although *D.ater*, *D.haemorrhoidalis*, *D.lardarius*, and *D.undulatus* have been reported from human cadavers (Charabidze et al. 2014; Kadej et al. 2020), none of the forensic entomological studies in Saudi Arabia reported any of them. As for *Marioutastangei* and *Rhopalosilphawasmanni*, no information is available that documents their feeding habits.

Despite more than 60 years since the first faunistic study (Shalaby 1961), we may still have an imprecise idea about the actual species number and faunal composition of Dermestinae in Saudi Arabia. In conclusion, the few numbers of faunistic studies on the Dermestinae in Saudi Arabia and the registration of two new records in the current study indicate that there are more species that have yet to be discovered.

## Supplementary Material

XML Treatment for Dermestes (Dermestes) ater

XML Treatment for Dermestes (Dermestes) haemorrhoidalis

XML Treatment for Dermestes (Dermestes) lardarius

XML Treatment for Dermestes (Dermestinus) frischii

XML Treatment for Dermestes (Dermestinus) maculatus

XML Treatment for Dermestes (Dermestinus) undulatus

XML Treatment for Mariouta stangei

XML Treatment for Rhopalosilpha wasmanni
